# Dyspepsia

**Published:** 1878-01-20

**Authors:** Fred. King

**Affiliations:** Atlanta, Ga.


### SOUTHERN MEDICAL RECORD.

VOL. VIII. ATLANTA, GA., JANUARY 20, 1878. NUMBER 1.

EDITORS:

THOMAS S. POWELL, M. D. W. T. GOLDSMITH, M. D. R. C. WORD, M. D.

*R. C. WORD, M. D., Managing Editor.*

SUBSCRIPTION—$2.00 PER ANNUM, IN ADVANCE.

☞ All Communications and Letters on Business connected with THE RECORD, for 1877 and 1878, must be addressed to the Managing Editor.

### *ORIGINAL AND SELECTED ARTICLES.*

### *DYSPEPSIA.*

BY FRED. KING, M.D., PH.G., Atlanta, Ga.

Notwithstanding the fact that a great deal has been written on dyspepsia, I think many physicians are almost entirely ignorant of its causes, and the many pathological conditions that are to be observed in the various forms of the disease. This is owing to the fact that a majority of us do not regard indigestion of sufficient importance to be enumerated among the serious maladies with which we have to deal. When consulted by dyspeptics, we are too prone to regard them as hypochondriacs, and to dismiss them after having prescribed a few bitter tonics and given some directions as to their diet. By such hasty and unjustifiable proceedings, we do ourselves injury and our patients injustice. If any relief is afforded, it is only temporary, and patients finally seek the "quack" and "stomach bitter men," who are ever ready to rob them of their last farthing.

Dyspepsia embraces all abnormal conditions of the digestive organs—stomach, liver, pancreas and small intestines; hence, the field is so broad that it is no easy task for us, when called upon to treat a patient suffering from indigestion, to reach a satisfactory diagnosis without thoroughly studying each individual case. We rarely meet with two consecutive cases in which the same symptoms are observable. This, of course, is owing to the fact that different organs connected with the functions of digestion are at fault. The symptoms in all cases are local; that is, they proceed directly from the stomach or small intestines—the first stages of digestion taking place in the former, the last in the latter. When the symptoms are traceable to the stomach, we may attribute them to deficiency in the quantity or quality of the gastric juice. It is impossible to enumerate, in a short paper like this, the many causes of this deficiency. We should always carry our investigations far enough to be able to locate, if possible, the exact seat of the trouble with which we have to deal, and then govern ourselves accordingly.

Under two main or principal heads may be classified all forms of dyspepsia: First, labored or difficult digestion; and second, symptoms of disturbed or imperfect digestion. A sense of discomfort is always the result of any kind of indigestion, while on the other hand, ease and comfort always follow the proper performance of this most important of all functions.

Dyspeptics are nearly always uneasy or miserable while the processes of digestion are going on; in many cases, amounting to the most extreme melancholy. These are oftentimes the only symptoms present. When such a state of things exist, we may at once conclude that we are to deal with labored or difficult digestion, resulting from a deficiency of some of the elements of the gastric juice, and must therefore prescribe such remedies as are calculated to make up for this deficiency. If the symptoms proceed from disturbed or imperfect digestion, they are traceable to a point lower down in the alimentary canal.

The most prominent symptoms of dyspepsia—such as regurgitations, cardialgia, catharsis tympanites, vomiting, etc., are so well known to us all that I deem anything like an extended reference to them superfluous. They all result from different causes—some originating in the stomach, while others come from a point below the pyloric orifice of that organ. A review of the many pathological changes that are to be observed in these abnormal conditions of the organs engaged in digestion, would be quite interesting, and indeed very conducive to a better understanding of the subject under consideration, did space permit. But I will hasten on to the consideration of those remedies which, in my opinion, are best fitted for the relief of *all forms* of dyspepsia.

In nearly all cases of indigestion, I am sure that the prime cause of the disorder may be found in the stomach or in the pancreas, and I am satisfied that the latter organ is as often at fault as the former. The symptoms in a large proportion of the cases that have come under my observation, point strongly to the truthfulness of this proposition. I am decidedly inclined to the opinion that many cases of supposed "chronic diarrhœa" are nothing but indigestion dependent upon a want of proper emulsification. The fats and oily substances passing through the intestines without being emulsified, I think, act as a mechanical cathartic, just as melted lard when swallowed in very large quantities. A careful examination of the fæces in each individual case would throw additional light on the diagnosis in such cases. Now, as we know that a certain amount of organic matter (pepsin and pancreatine), lactic acid, chlorides of soda, potash, lime and ammonia, muriatic acid, etc., is indispensible in the process of digestion, assimilation and nutrition, it necessarily follows that impairment of these functions depends on a deficiency of some one or more of these elements. We should, therefore, prescribe such preparations as are calculated to restore these lost agents. In my experience nothing has given such *entire* satisfaction as *lactopeptine*, which is a combination of sugar of milk, pepsin, pancreatine, diastase, lactic and hydrochloric acids scientifically combined by experienced and competent chemists.* In this combination we have all the agents concerned in digestion from the time food is masticated until it is converted into chyle. My employment of the remedy in various forms of indigestion during the last year, was founded upon chemical observations in regard to the power of *lactopeptine* as a digestive agent. I found by actual experiment, its digestive power to be more than five times as great as any preparation of pepsin or pancreatine that I could obtain in our city. These experiments were made upon meats of various kinds, bread, fats, etc., or about what usually constitutes a meal for an ordinary adult. I tried its force on coagulated

* Reed & Carnrick, New York.

albumen as well as fats, and found its powers far superior to those of anything I had before experimented with. I made these experiments for the gratification of my own curiosity, regardless of the fact that the preparation had been highly praised and extensively prescribed by such men as Leaming, Loomis, Simms, Janeway and Tyndall, of New York, when it was first introduced.

One of the most important among the many advantages possessed by lactopeptine, over other medicines recommended for indegestion, is that it contains the natural acid secreted by the stomach, viz: lactic and hydrochloric, without which the character of coagulated albumen nor fatty substances, is not changed by pepsin or pancreatine. Pepsin will not emulsify, nor will pancreatine desolve coagulated albumen, but lactopeptine will do both.

And before reporting, or rather furnishing a synopsis of a few cases treated, where lactopeptine was prescribed, I will give my reason for diminishing the dose from time to time. I did so from the fact that a certain amount of the elements contained in the preparation was absorbed, and of course found their way through the medium of the circulation back to the stomach, pancreas, etc., and again aided in the work they were first intended to assist.

Case 1.—Ex-Governor C. consulted me sometime in September last, suffering from dyspepsia of long standing; symptoms pointing mainly to pyloric orifice of stomach, I gave him the following: R.—Lactopeptine, dr. iij.; vin. xerice, oz. iij. M—Sig. tablespoonful after each meal, gradually reducing it to a teaspoonful. At expiration of one month he expressed himself as being *entirely* relieved.

Case 2.—Mr. S. T. D., ice dealer, had been suffering from diarrhœa for more than four weeks. He applied to me in August. After a careful diagnosis I pronounced his trouble indigestion. The lactopeptine given the same way as case one, cured him in a week.

Case 3.—Mrs. P. H. T., aged 38, anemic, suffering from cordialgia, and also vomiting, was pregnant six months. I gave her, R. eilx. lactopeptine with bark and iron, oz. vi. S. dessertspoonful after meals. She was entirely relieved after a fortnight.

Case 4.—A little daughter of Dr. W. C., dentist, had but little appetite, and spat up what food she attempted to eat. I prescribed, with the happiest results, R—Syr. Lactopeptine comp. oz. ij.; S—teaspoonful after each meal, gradually reduced to ten drops.

Case 5.—Dr. A., a professional friend, had suffered for more than two years from neuralgia, which he attributed to indigestion. Lactopeptine, prescribed in same form as case one, relieved him.

I find that different patients require different forms of the preparation. While adult males take the dry powder, liquid or wine, females and children must have the elixir or syrup. It can be obtained in combination with the leading ferruginous and bitter tonics, cod liver oil, bismuth, strychnine, lime, etc., meeting in such a variety of preparation all forms and complications of dyspepsia.

